# Modulation of Intestinal Microbiota by the Probiotic VSL#3 Resets Brain Gene Expression and Ameliorates the Age-Related Deficit in LTP

**DOI:** 10.1371/journal.pone.0106503

**Published:** 2014-09-09

**Authors:** Eleonora Distrutti, Julie-Ann O’Reilly, Claire McDonald, Sabrina Cipriani, Barbara Renga, Marina A. Lynch, Stefano Fiorucci

**Affiliations:** 1 S.C. di Gastroenterologia ed Epatologia, Azienda Ospedaliera di Perugia, Perugia, Italy; 2 Trinity College Institute for Neuroscience, Department of Physiology, Trinity College, Dublin, Ireland; 3 Dipartimento di Medicina, Università degli Studi di Perugia, Perugia, Italy; 4 Dipartimento di Scienze Chirurgiche e Biomediche, Università degli Studi di Perugia, Perugia, Italy; Tokai University, Japan

## Abstract

The intestinal microbiota is increasingly recognized as a complex signaling network that impacts on many systems beyond the enteric system modulating, among others, cognitive functions including learning, memory and decision-making processes. This has led to the concept of a microbiota-driven gut–brain axis, reflecting a bidirectional interaction between the central nervous system and the intestine. A deficit in synaptic plasticity is one of the many changes that occurs with age. Specifically, the archetypal model of plasticity, long-term potentiation (LTP), is reduced in hippocampus of middle-aged and aged rats. Because the intestinal microbiota might change with age, we have investigated whether the age-related deficit in LTP might be attenuated by changing the composition of intestinal microbiota with VSL#3, a probiotic mixture comprising 8 Gram-positive bacterial strains. Here, we report that treatment of aged rats with VSL#3 induced a robust change in the composition of intestinal microbiota with an increase in the abundance of *Actinobacteria* and *Bacterioidetes*, which was reduced in control-treated aged rats. VSL#3 administration modulated the expression of a large group of genes in brain tissue as assessed by whole gene expression, with evidence of a change in genes that impact on inflammatory and neuronal plasticity processes. The age-related deficit in LTP was attenuated in VSL#3-treated aged rats and this was accompanied by a modest decrease in markers of microglial activation and an increase in expression of BDNF and synapsin. The data support the notion that intestinal microbiota can be manipulated to positively impact on neuronal function.

## Introduction

Age-related changes in the brain contribute to the time-related deterioration in cognitive function. These include neuroinflammatory and oxidative changes with the associated glial activation, as well as loss of synaptic connections and perhaps neurons, and reduced neurogenesis [1]. The deterioration in cognitive function is manifest by poorer spatial learning [2], while the alterations at the level of the synapse are reflected by loss of plasticity, for example a poorer ability of animals to sustain long-term potentiation (LTP). Significantly, when these age-related neuroinflammatory changes are attenuated by treating aged rats with polyunsaturated fatty acids [3], statins [4] or a combination of vitamin D and dexamethasone [5], [6], LTP is partially restored. Similarly, the expression of brain-derived neurotrophic factor (BDNF), which induces neurogenesis [7] and enhances the ability of rats to sustain LTP, positively correlates with LTP [8]. Additionally, an increased expression of hippocampal BDNF has been associated with restoration of LTP in middle-aged rats [9].

Whereas the brain is protected to a significant degree by the existence of the blood brain barrier, it has become clear that it is not the immune-privileged organ that was considered in the past [10]. Indeed peripheral infections have been known for many years to impact on neuronal function and the CNS effects of sickness behaviour have been well rehearsed [10]. Peripheral administration of lipopolysaccharide increases inflammatory and oxidative stress in brain, and glial activation in the hippocampus, to a greater extent in aged, compared with young, rats [11]. Consistently, age is associated with increased vulnerability to infections, while infections have been shown to accelerate progression of diseases such as Alzheimer's disease and multiple sclerosis [12], [13]. These findings support the notion that a communication network between the CNS and the periphery exists. This communication network is consolidated by the association between psychiatric conditions, like anxiety, and the inflammatory changes in the gastrointestinal tract that typify inflammatory bowel disease [14].

A reciprocal interaction exists between the gut microbiota and CNS function [15]. For example, it has been shown that the stress associated with neonatal maternal separation induces cognitive dysfunction and impacts on composition of gut microbiota [16], [17], although the effects change with the time and duration of separation [18], [19]. Similarly, mice kept in germ-free conditions, in which development of the hypothalamic-pituitary-adrenal axis is impaired, exhibit deficits in cognitive function [20]. Interestingly, stress negatively impacts on cognitive function in mice infected with *Citrobacter rodentium*, but this deficit is normalized if animals are pretreated with probiotics [20], which effectively attenuated inflammatory changes in the colon and infection-induced decrease in hippocampal BDNF.

Anti-inflammatory effects of the probiotic strains, *Lactobacillus rhamnosus* [21] and *Lactobacillus reuteri* [22] and VSL#3 [23] have been reported. VSL#3 is a mixture of 8 different strains of bacteria, namely *Streptococcus thermophilus* DSM24731, *Bifidobacterium breve* DSM24732, *Bifidobacterium longum* DSM24736, *Bifidobacterium infantis* DSM24737, *Lactobacillus acidophilus* DSM24735, *Lactobacillus plantarum* DSM24730, *Lactobacillus paracasei* DSM24733, *Lactobacillus delbrueckii subspecies Bulgaricus* DSM24734. VSL#3 administration to mice rendered colitic by exposure to the barrier-braking agent sodium dextran sulphate, attenuated changes in COX2, iNOS, TNFα and IL-6 in the colon [24]. This effect has been linked to the ability of the probiotic mixture to increase IL-10 [25]. Further, VSL#3 has been shown to reduce pain in an animal model of visceral hypersensitivity (i.e. the hyperalgesia induced by neonatal maternal separation) and this was associated with treatment-induced changes in expression of genes encoding for proteins involved in nociception and inflammation [26], further emphasizing a modulatory role for VSL#3 in stressful conditions. Clinical studies have confirmed the VSL#3 is effective in reducing inflammation and symptoms in clinical settings [27], [28].

In the present study, we set out to assess whether treatment with VSL#3 modulates neuronal functions and LTP in young and aged rats. We found that the age-related deficit in LTP was markedly attenuated in rats receiving VSL#3, while gene array analysis revealed that the probiotic mixture resets the expression of several genes in the brain.

## Materials and Methods

### Animals

Young (3 months; 250–350 g) and aged (20–22 months; 550–600 g) male Wistar rats (Bantham and Kingman, UK) were housed in a controlled environment (temperature: 20–22°C; 12∶12 h light/dark cycle) in the BioResources Unit, Trinity College, Dublin. Young and aged rats were subdivided into 2 groups: those which were given VSL#3 (CD Investments s.r.l, Rome, Italy) at the dose of 12.86 bn living bacteria/kg/day [29] in maple syrup (90–160 µl) for 6 weeks [young VSL#3-treated (YV) and aged VSL#3-treated (AV)] and control rats which received only maple syrup [young control-treated (YC) and aged control-treated (AC)]. Experiments started at day 0 and ended after 6 weeks (day 42). Rats had free access to food and water and were maintained under veterinary supervision for the duration of the experiment. All experiments were carried out under licence from the Department of Health and Children (Ireland) and with ethical approval from Trinity College Ethical Committee.

### Analysis of intestinal microbiota: DNA extraction, amplification, digestion and fragment sizing

To collect stool samples at the beginning (day 0) and end of the treatment period (day 42), rats were individually placed in a clean cage separated with paper and faecal pellets were placed in individual tubes and stored at −80°C. Samples were shipped from Trinity College Dublin to the University of Perugia on dry ice. DNA was extracted from 200 mg of frozen stool samples using the QIAamp DNA Stool mini Kit (Qiagen) according to manufacturer's instructions. Primers 8f (AGAGTTTGATCCTGGCTCAG) and 536r (GWATTACCGCGGCKGCTG) were applied to 200 ng DNA in order to amplify a part of the 16S rRNA using the PCR protocol for Phusion High Fidelity DNA Polymerase (New England Biolabs). Forward primers were fluorescently labeled (WellRED D4dye, Sigma-Proligo, St. Louis, MO) to allow detection of the fragments by capillary electrophoresis. The polymerase chain reaction (PCR) was as follows: 98°C for 30′′; 40 cycles at 98°C for 10′′; 61°C for 30′′; 72°C for 30′′; and a final extension at 72°C for 10 minutes. The PCR product (∼528 base pairs) was purified using the QIAmp PCR purification kit (Qiagen). To produce terminal restriction fragments (T-RF) 10 µl of PCR product was digested using the restriction enzyme Hha I (New England Biolabs). The mix was adjusted to a final volume of 20 µl with DNase/RNase-free water and the DNA was digested at 37°C for 4 hours. The precise length of T-RF amplicons was determined by performing capillary gel electrophoresis with a CEQ 8000 Genetic Analysis System (Beckman Coulter). Four µl of fluorescently labeled fragments, 35.5 µl of sample loading solution (SLS) (Beckman Coulter) and 0.5 µl of 600 bp DNA standard size (Beckman Coulter) were mixed and separated using the frag4 protocol. An electropherogram with peaks of different size was obtained for each stool sample. Fragment analysis was performed using CEQ software version 9.0.

### Bioinformatic analysis of T-RFLP data

MiCA (Microbial Community Analysis III) on-line software (http://mica.ibest.uidaho.edu/about.php) was used to build a putative reference database of T-RF of the gut. The analysis was performed using as reference the H.Q. database. Primers 8f and 536r, Hha I restriction enzyme and T-RFLP data obtained from CEQ software (Fragment sizes, migration time and peak area) were applied to PAT (Phylogenetic Assignment tool of MiCA) and a library of probable species was obtained for each sample. Almost all species found in this study belong to the four most populated bacterial phyla, namely *Bacteroidetes, Proteobacteria, Firmicutes*, and *Actinobacteria*. Thus, values reported in this analysis are expressed as percentage of these four phyla.

### Microarray analysis

According to Data Availability Statement of PlosOne, the data discussed in this publication have been deposited in NCBI's Gene Expression Omnibus (GEO) [30] and are accessible through GEO Series accession number **GSE51381**. All microarray analysis were performed by MiltenyiBiotec, GmbH Bioinformatics, German. Microarray analysis was performed on cortical samples from young control-treated rats (YC), young VSL#3-treated rats (YV), aged control-treated rats (AC), aged VSL#3-treated rats (AV).

### Preparation, amplification and hybridization of RNA

RNA was isolated from rat tissue samples by using standard RNA extraction protocols (Trizol) and the RNA was quality-checked via the Agilent 2100 Bioanalyzer platform (Agilent Technologies). Four replicates were assessed per sample. For the linear T7-based amplification step, 100 ng of each RNA sample was used. To produce Cy3-labeled cRNA, the RNA samples were amplified and labelled using the Agilent Low Input Quick Amp Labeling Kit (Agilent Technologies) following the manufacturer's protocol. Yields of cRNA and the dye-incorporation rate were measured with the ND-1000 Spectrophotometer (NanoDrop Technologies).

The hybridization procedure was performed according to the Agilent 60-mer oligo microarray processing protocol using the Agilent Gene Expression Hybridization Kit (Agilent Technologies). Briefly, 0.6 µg Cy3-labeled fragmented cRNA in hybridization buffer was hybridized overnight (17 hours, 65°C) to Agilent Whole Rat Genome Oligo Microarrays 8×60K using Agilent's recommended hybridization chamber and oven. Fluorescence signals of the hybridized Agilent Microarrays were detected using Agilent's Microarray Scanner System (Agilent Technologies).

### Image and data analysis

The Agilent Feature Extraction Software (FES) was used to read out and process the microarray image files. The software determines feature intensities (including background subtraction), rejects outliers and calculates statistical confidences. For determination of differential gene expression FES derived output data files were further analyzed using the Rosetta Resolver gene expression data analysis system (Rosetta Biosoftware). All samples were labelled with Cy3. The ratio experiments are designated as control versus (vs) sample experiments (automated data output of the Resolver system) with the ratios calculated by dividing sample signal intensity through control signal intensity.

The bioinformatics data analysis of eleven microarray datasets obtained from one-colour hybridization of rat RNAs on Agilent Whole Rat Genome Oligo Microarrays 8×60K was performed. Pre-processing of the data, including normalization and correlation analysis, was followed by differential gene expression analysis (DGA) for AC versus YC, AC versus AV, YC versus YV and YC versus AV. These analyses aimed to distinguish changes in expression among the four groups of samples so that eight discriminatory gene sets (for each group up- and downregulated genes) were analyzed. A combination of statistical methods and the magnitude of expression difference (fold change) were applied in order to identify genes with differential expression between two sample groups. For the detection of discriminatory expression, genes were selected that show a statistically significant deviation in the test compared with the reference group (ANOVA p-value ≤0.05, Tukey p≤0.05). Average expression value was at least 1.5-fold higher or lower than the reference average. To enable construction of a red/green heatmap, the expression values were converted to “virtual ratios” by referencing each individual intensity signal to median of all intensities. The base-2 logarithms of these virtual ratios were used to prepare the heatmap display. A comparison of the discriminatory gene sets among different groups is shown using Venn diagrams.

### Microarray validation by PCR

Quantification of the expression of selected genes was performed by quantitative real-time PCR (qRT-PCR). 1 µl of the remaining RNA from cortex samples that were used for gene array was incubated with DNase I and reverse-transcribed with Superscript II (Invitrogen) according to manufacturer specifications. For real-time PCR, 10 ng of template was used in a 20µl reaction containing 0.2 µM of each primer and 10 µl of KAPA SYBR FAST (KapaBiosystem). PCR primers were designed using the software PRIMER3-OUTPUT using published sequence data obtained from the NCBI database.

rPLA2G3 s: gcaccaacgaaggagaagag

rPLA2G3 as: gcaagggtgagatggtttgt

rAlox15s: tacctgtggttggttggaca

rAlox15as: ggcgtcatccgtgagataat

rNid2s:gccttcagagccagatgttc

rNid2as:ggtcctccagtcctaccaca

### Analysis of LTP in vivo

At the end of the 6 week period during which animals received VSL#3 in maple syrup or maple syrup alone, rats were anaesthetized with urethane (1.5 g/kg urethane intraperitoneally) and assessed for their ability to sustain LTP as previously described [31]. Briefly, when rats reached a state of deep anaesthesia, verified by the absence of a pedal reflex, they were placed in a stereotaxic frame and a bipolar stimulating electrode was slowly lowered into the perforant path (4.4 mm lateral and 8.0 mm posterior to Bregma) and a unipolar recording electrode was slowly lowered into the dorsal cell body region of the dentate gyrus (2.5 mm lateral and 3.9 mm posterior to Bregma). The depth of the electrodes was adjusted in the cell body region to obtain potentials with maximum amplitude, and the stimulus strength was chosen to ensure that a population spike of approximately 1 mV was evident. Test shocks were delivered at 30 s intervals for up to 1 hour to establish a stable baseline and, after this time, recordings were collected for 15 minutes prior to delivery of 3 trains of high-frequency stimuli (250 Hz for 200 ms; 30 s inter-train interval). Recording at test shock frequency resumed for the remaining 45 minutes of the experiment. The slope of the excitatory post-synaptic potential (epsp; mV/ms) was used as a measure of excitatory synaptic transmission in the dentate gyrus. At the end of the experiment, rats were killed by cervical dislocation, the hippocampus and cortex dissected free and snap frozen for later analysis.

### RT-PCR and Western blot analysis from hippocampal tissue

Total RNA was extracted from snap-frozen hippocampal tissue using a NucleoSpin RNAII isolation kit (Macherey-Nagel Inc., Germany) as described by the manufacturer. RNA integrity and total RNA concentration were assessed, and cDNA synthesis was performed as described previously [32]. Real-time PCR was performed using Taqman Gene Expression Assays (Applied Biosystems, Germany) which contain forward and reverse primers, and a FAM-labeled MGB Taqman probe for each gene of interest. The assay IDs for the genes examined in this study were as follows: CD11b (Mm001271265_m1), CD68 (Rn01495631_g1), GFAP (DETAILS), BDNF (DETAILS and NGF (DETAILS). Each well contained 20 µl comprising diluted cDNA (9 µl), primer (1 µl) and Taqman Universal PCR Master Mix (10µl) and samples were assayed in duplicate. Each run (40 cycles) consisted of 3 stages, 95°C for 10 min, 95°C for 15 sec for each cycle (denaturation) and finally the transcription step at 60°C for 1 min. The endogenous control, used to normalize gene expression data, was β-actin and β-actin expression was conducted using a gene expression assay containing forward and reverse primers (primer limited) and a VIC-labeled MGB Taqmanprobe (Applied Biosystems, Germany; Assay ID: 4352341E). Gene expression was calculated relative to the endogenous control samples and to the control sample giving an RQ value (2^−DDCt^, where CT is the threshold cycle).

For Western blot analysis, hippocampal homogenates were prepared in lysis buffer as described [33]. Lysates were centrifuged (20000 x g, 12 min) and supernatant samples (10 µg) were added to NuPAGE LDL sample buffer, heated at 70°C for 10 min and separated on 4–12% gradient gels (Invitrogen, UK). Proteins were transferred to nitrocellulose membrane (Sigma, UK) and blocked for 1 hour in Tris-buffered saline-0.05% Tween-20 (TBS-T) and 5% bovine serum albumin (BSA). Membranes were incubated overnight at 4°C with anti-synaptophysin (1∶5,000; Sigma, UK) in TBS-T/1% BSA, washed and incubated with secondary antibody (1∶5000 in 5% BSA/TBS-T; Sigma, UK) for 2 h. Immunoreactive bands were detected using enhanced chemiluminescence (Amersham, UK) and blots were stripped (Re-blot Plus; Chemicon International, Temecula, CA) and re-probed using anti-β-actin (1∶4000 in 5% BSA/TBS-T; Sigma, UK) and a peroxidase conjugated secondary antibody (1∶1000 in 5% BSA/TBS-T; Sigma, UK). Bands were quantified by densitometry (Labworks v4.5, MediaCybernetics, Bethesda, MD). Values were normalized for protein loading using the actin protein expression values.

### Statistical analysis

Data were analyzed using either Student's t-test for independent means, or analysis of variance (ANOVA) followed by post hoc Student Newman–Keuls test or Tukey test to determine which conditions were significantly different from each other. Data are expressed as means with standard errors.

For microarray analysis a combination of statistical methods (ANOVA p-value ≤0.05, Tukey p≤0.05) and the magnitude of expression difference (1,5-fold change higher or lower than the reference average) was applied.

## Results

### Analysis of intestinal microbiota composition

**Figure 1:**
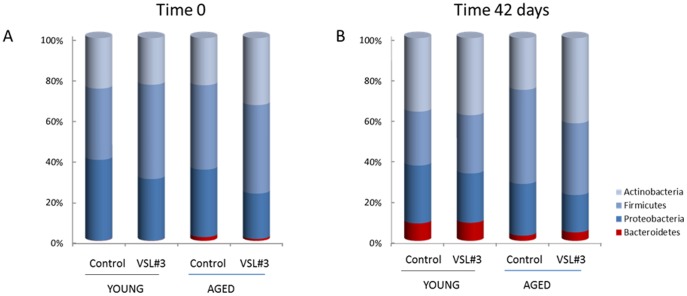
Phylogenetic profiles of gut microbiote in young and aged rats treated with a control diet or VSL#3. (A-B). Terminal restriction fragments (T-RFs) of microbial communities from feces of young control rats (YC), young rats administered VSL#3 (YV), aged control rats (AC) and aged rats administered VSL#3 (AV) from day 0 (panel A) and 42 (panel B)were assigned to hierarchical taxonomic groups using the on-line software MiCA. Values of taxonomic ranks are expressed in percentage as a proportion of the reference library used for analysis (H.Q. database).

At day 0, young control-treated rats (YC) and young rats treated with VLS#3 (YV) showed only 3 of the 4 most populated bacterial phyla, as *Bacteroidetes* were not detected (Figure 1A). In YC at day 0, *Proteobacteria* was the most representative bacterial family (39.8±3.3%), followed by *Firmicutes*, (35.1±2.0%) and *Actinobacteria* (25±3.9%). Noteworthy, at day 0 there was no significant difference in bacterial composition of YV in comparison with YC, as *Bacteroidetes* were not detected, while *Firmicutes* represented 47±4.6% of the total, followed by *Actinobacteria* (30±6.8%) and *Proteobacteria* (23±2.3%) (Figure 1A). In samples from YC at day 42, the bacterial composition spontaneously changed, as *Bacteroidetes* appeared (9±1.8% of the total; p<0.05 versus day 0), while a trend towards an increased percentage of *Actinobacteria* (36±8.8%), and a decreased percentage of both *Firmicutes* and *Proteobacteria* was observed (27±3.2% and 28±8% respectively) (Figure 1B). At day 42, samples from YV showed the same microbiota composition as that of YC, as *Bacteroidetes and Actinobacteri*a phyla increased (9±2.7% and 38±11% respectively) while both *Firmicutes and Proteobacteria* decreased (29±1.7% and 24±9.1% respectively) (Figure 1B). These results indicate that microbiota spontaneously changed during the 6 week period of the experiment, and this effect was independent of VSL#3 administration.

At day 0, the percentage of the 4 main bacterial families was similar in the aged control-treated rats (AC) and aged VSL#3-treated rats (AV), but the *Bacteroidetes* phylum was significantly greater in AC in comparison with YC (p<0.05; Figure 1A). Microbiota composition did not significantly change between day 0 and day 42 in AC rats, while in AV there was a significant increase in *Bacteroidetes* phylum at day 42 in comparison with day 0 (p<0.05; Figure 1A and B). Furthermore, in AV there was a trend in the increase of *Actinobacteria* and a reduction of *Firmicutes* between day 0 and day 42 (Figure 1A and B).

### Global microarray analysis of brain tissue

**Figure 2:**
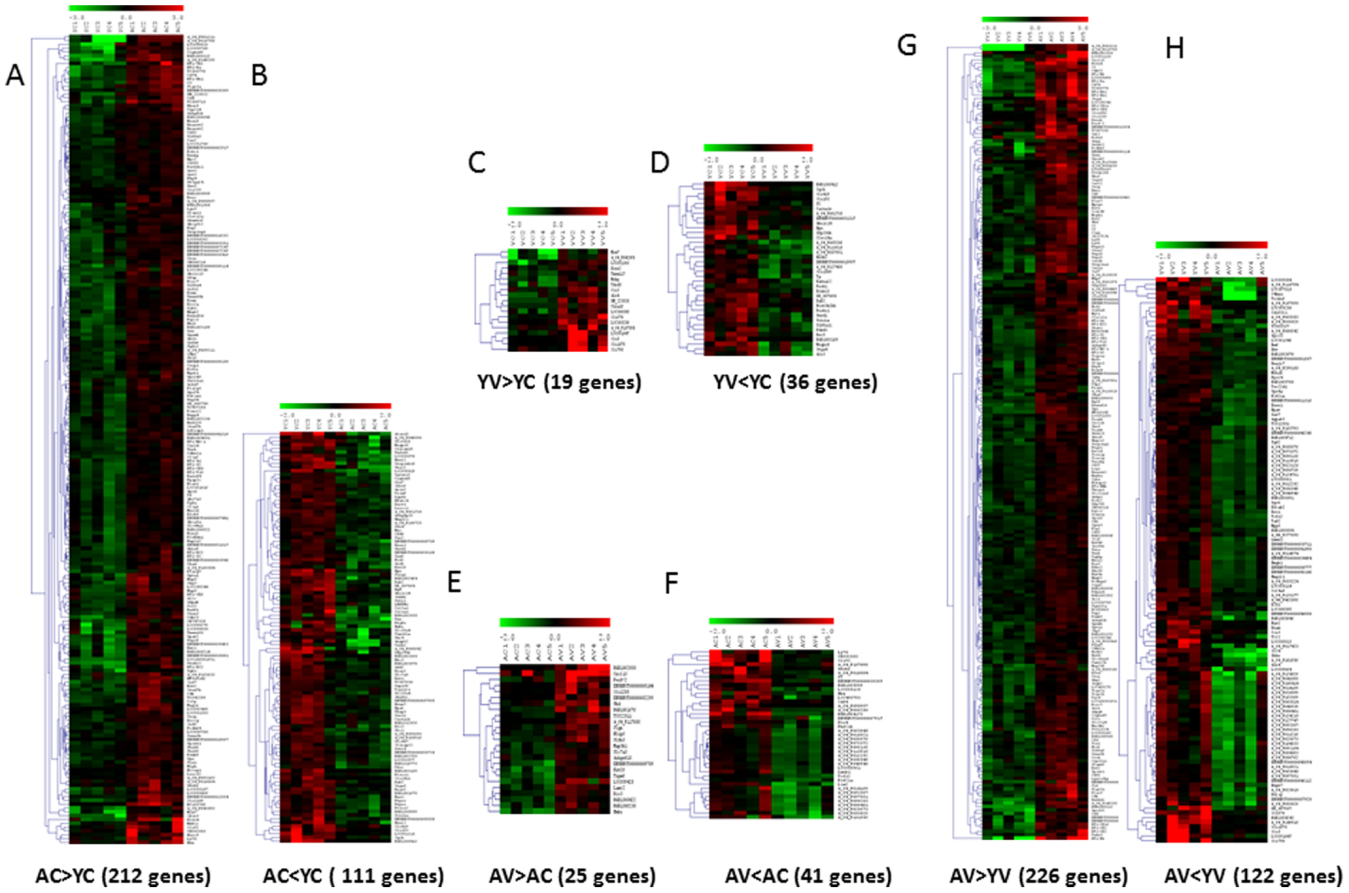
Microarray analysis of genes in cortical tissue of young and aged rats shows modulation by age and VSL#3. (A–B) Heat maps of the significantly regulated genes (p<0.05 and 1.5-fold up- or downregulation) in cortical tissue of control-treated aged rats (lanes AC1–AC5) in comparison with control-treated young rats (lanes YC1-YC5). We detected changes in the expression of 323 genes (about 1% of total). Thus, 212 were upregulated and 111 downregulated. VSL#3 treatment effectively modulates the expression of genes in cortical tissue of both young (panel C and D for upregulated and downregulated genes respectively) and aged (panel E and F for upregulated and downregulated genes respectively) rats. Comparison between YV and AV demonstrates that more that 300 genes are significantly modulated (panel G and H for upregulated and downregulated genes respectively). Microarray data from 5 rats are presented.

**Figure 3:**
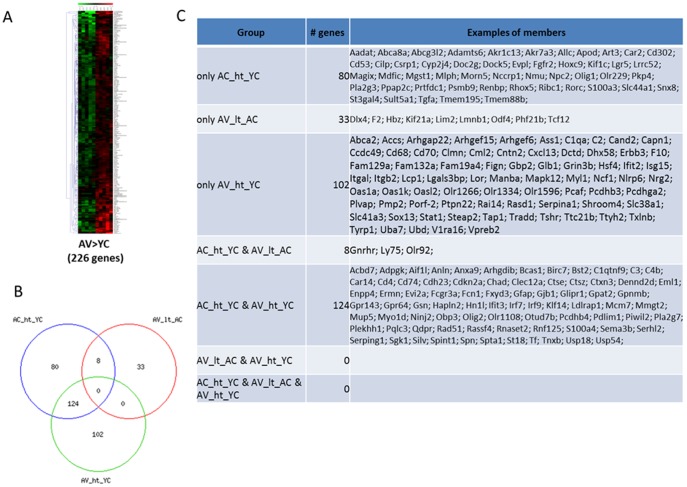
Microarray analysis of genes in cortical tissue of young and aged rats shows modulation by age and VSL#3. (A) Heat maps analysis of changes caused by VSL#3 administration in VSL#3-treated aged rats (lanes AV1-AV5) in comparison with control-treated young rats (lanes YC1-YC5) demonstrate that expression of 226 genes was modulated >1.5-fold (p<0.05). (B) Venn diagram of modulated genes indicates that age was associated with an upregulation of 226 genes;8 of these genes were downregulated by the probiotic treatment. (C) The table presents the complete list of all genes that were significantly modulated in control-treated aged rats in comparison with control-and VSL#3-treated young rats and VSL#3-treated aged rats. Microarray data from 5 rats are presented. ht: higher than; lt: lower than.

**Figure 4:**
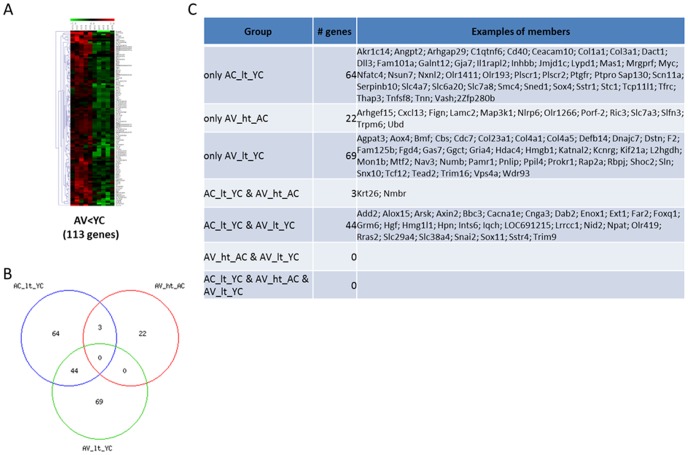
Microarray analysis of genes in cortical tissue of young and aged rats shows modulation by age and VSL#3. (A) Heat maps analysis of changes caused by VSL#3 administration in VSL#3-treatedagedrats (lanes AV1-AV5) in comparison with control-treated young rats (lanes YC1-YC5) demonstrate that expression of 113 genes was modulated <1.5-folds (p<0.05). (B) Venn diagram of modulated genes shows that age was associated with a downregulation of 113 genes;3 of these genes were upregulated by the probiotic treatment. (C) The table presents the complete list of all genes that were significantly modulated in control-treated aged rats in comparison with control- and VSL#3-treated young rats and VSL#3-treated aged rats. Microarray data from 5 rats are presented. ht: higher than; lt: lower than.

Global microarray analysis, performed on cortical tissue obtained from the rats in each of the 4 groups, AC, YC, AV, and YV, screened 30,367 genes and detected several differentially-expressed genes (data are available at website http://www.ncbi.nlm.nih.gov/geo/query/acc.cgi?token=kjirwsoqzlapbit&acc=GSE51381). When the combination of statistical methods (p-value <0.05) and the magnitude of gene expression difference (fold change at least ±1.5) was applied, 333 genes (1.1% of total) were found to be significantly modulated in aged rats compared with young rats (Figures 2–4). The hierarchical clustering of these genes provided good separation based on the expression of the genes in the four groups of rats. In Figures 2–4, red and green colours indicate upregulated and downregulated genes respectively, while black colour indicates those genes whose expression was not changed. As shown in Figures 2A and 2B, when the pattern of gene expression in the brain cortex of aged control-treated rats (lanes AC1-AC5) was compared with that of young control-treated rats (lanes YC1-YC5), we detected a significant up- and downregulation of 212 and 111 genes, respectively. The complete list of these genes is available at the above mentioned website. We have then investigated whether VSL#3 could modulate these age-associated changes. To this end, the pattern of gene expression in the brain cortex of aged rats treated with VLS#3 (lanes AV1-AV5) was compared with the pattern of gene expression in tissues obtained from control-treated young rats (lanes YC1-YC5). The results of this comparison are shown in Figure 3 and 4. We found that the two animal groups differ for expression of 339 genes, 226 of which were significantly upregulated (Figure 3A) while 113 were downregulated (Figure 4A). The effect of VSL#3 was further analysed by using Venn diagrams which demonstrate that 8 genes which were upregulated in AC in comparison with YC, were then downregulated by VLS#3 treatment (AV, Figure 3B), while 3 genes that were downregulated in AC in comparison with YC were then upregulated by VLS#3 treatment (AV, Figure 4B), indicating that the probiotic intervention at least partially reversed the effects of aging on these genes. The list of these genes is shown in Figure 3C and 4C.

Further analysis of 226 genes that were upregulated in AV in comparison with YC, demonstrated that 102 new genes were induced by VSL#3 treatment *per se* (Figure 3B). Similarly, among the 113 genes that were downregulated in AV in comparison with YC, 69 were new genes specifically modified by administering aged rats with VSL#3 (Figure 4B).

The effect of probiotic treatment was further investigated by comparing the pattern of gene expression in the cortical tissues obtained from both young and aged rats treated or not treated with the VSL#3. Of relevance, we found that the probiotic diet *per se* effectively modulated the expression of several genes in both groups. This effect was downregulatory in nature as demonstrated by the fact that, while VSL#3 administration induced a significant modification in expression of 55 genes in YV in comparison with YC, 19 genes were upregulated and 36 genes were downregulated (Figure 2C and D respectively). Consistently, among the 66 genes whose expression was modulated by administering aged rats with VSL#3, 25 were upregulated while 41 were downregulated (AV vs AC; Figure 2E and F and Figure 3B and 4B respectively).

Finally, the effect of VSL#3 administration was further analysed by comparing the effect of VSL#3 administration to aged and young rats. This comparison demonstrate that young and aged rats administered VSL#3 differ in the expression of 338 genes, 226 of which were upregulated and 122 genes were significantly downregulated (Figure 2G and H respectively).

PCR analysis performed on a subset of genes strongly involved in neurodegenerative processes confirmed the microarray data for Alox15 gene that was downregulated in AC in comparison with YC (p<0.05 versus YC; Figure S1B) and PLA2G3 gene that was overexpressed in AC and return to levels of YC with probiotic diet (p<0.05 versus YC; p<0.05 versus AC; Figure S1C).

### Analysis of LTP and markers of inflammation in the hippocampus

**Figure 5:**
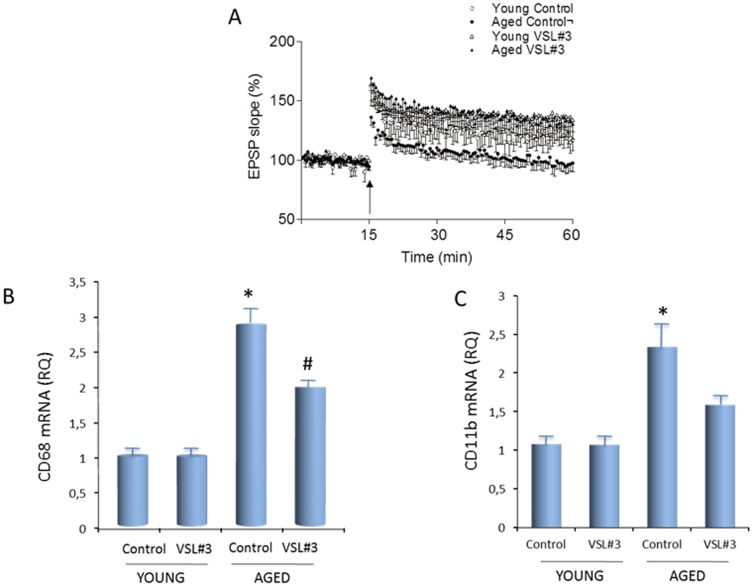
VLS#3 treatment attenuates the age-related decrease in LTP and the age-related increase in microglial activation. (A) Delivery of a high frequency train of stimuli to the perforant path (arrow) induced an immediate and sustained increase in EPSP slope in control-treated young rats and this effect was markedly decreased in control-treated aged rats. Aged rats treated with VLS#3 sustained LTP in a manner similar to young rats. (B, C) Expression of CD68 mRNA and CD11b mRNA was significantly increased in hippocampal tissue prepared from control aged rats compared with control young rats (*p<0.05 AC vs YC). Expression of both markers was reduced in VSL#3-treated aged rats compared with control aged rats, and the difference was statistically significant onlyin the case of CD68 mRNA (#p<0.05 AV vs AC).

To evaluate whether VLS#3 treatment exerted any effect on synaptic plasticity, we assessed the effect of delivery of a high frequency train of stimuli to the perforant path on changes in the dentate gyrus. The high frequency train of stimuli induced an immediate and sustained increase in epsp slope in YC and YV; the mean percentage increases in epsp slope in the last 10 minutes of the experiment in these 2 groups of rats were 122.2 (±0.92, SEM) and 136.3 (±0.37, SEM). The change in epsp slope following high frequency stimulation in AC was significantly reduced and the mean percentage change in the last 10 minutes of the experiment was 98.0 (±0.56, SEM). In contrast, AV sustained LTP in a manner similar to the YC and the mean percentage change in epsp slope in the last 10 minutes of the experiment in AV was 132.2 (±0.69, SEM; Figure 5A).

Previous data have indicated that the age-related deficit in LTP is associated with microglial activation and therefore we assessed expression of 2 markers of activation, CD68 mRNA and CD11b mRNA. The data show a significant effect of age in each marker (p<0.05 AC vs YC; Figure 5B and C). CD68 mRNA and CD11b mRNA were reduced in AV compared with AC but this difference reached significance only in the case of CD68 mRNA (p<0.05 AV vs AC; Figure 5B and C).

**Figure 6:**
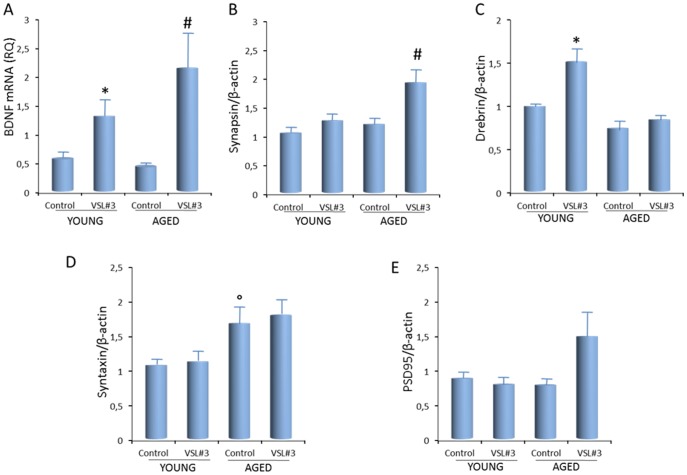
VLS#3 treatment increases BDNF and modulates the age-related changes in synaptic proteins. (A) VLS#3 treatment significantly increased expression of BDNF mRNA in hippocampal tissue(*p<0.05 YV vs YC; #p<0.05 AV vs AC). (B) A significant VSL#3 treatment effect on synapsin was observed in aged rats (#p<0.05 AV vs AC). (C) A significant treatment effect was observed in drebrin in young rats (*p<0.05 YV vs YC). (C) A significant age effect was observed in syntaxin (°p<0.05 AC vs YC). (E) No effect in PSD95 was observed.

Impaired LTP has also been associated with decreased BDNF expression and with decreased expression of synapic proteins and therefore we assessed BDNF mRNA in tissue prepared from these rats showing a significant VSL#3 treatment effect in both young and aged rats (p<0.05 YV vs YC; p<0.05 AV vs AC; Figure 6A). In addition, a significant VSL#3 treatment effect on synapsin was observed in aged rats (p<0.05 AV vs AC; Figure 6B), whereas a significant treatment effect was observed in the postsynaptic protein drebrin in young rats (p<0.05 YV vs YC; Figure 6C). We also observed a significant age effect in syntaxin (p<0.05 AC vs YC; Figure 6D), but no change in PSD95 was observed (Figure 6E). These data suggest that VSL#3 may have a synaptotrophic effect driven by BDNF.

## Discussion

A growing body of evidence supports the suggestion that the gut microbiota might have an effect on normal human behavior and that alterations in its composition or metabolism can play a role in the pathophysiology of psychiatric and neurologic diseases and on neuronal function which is impaired with age.

In the present study comparison between young and aged animal allows us to investigate whether age influenced intestinal microbiota composition, brain functions and brain gene expression and whether treatment of aged rats with the probiotic VSL#3 attenuated these alterations. By bioinformatic approach we have made a wide array of comparisons among all groups of animals including adult control rats and adult rats administered with VSL#3 (i.e. AV vs AC), but also we have arnessed to investigate whether the probiotic treatment reversed the age-induced alterations of brain functions by comparing this group with young control rats (i.e. AV versus YC). Although the latter comparison might be less circumstantial, the data shown in Figures 3–6 strongly support the notion that age related changes might be attenuated by the probiotic intervention, which also ameliorates the age-related deficit in LTP, a commonly-used method for assessing synaptic plasticity.

The composition of gut microbiota changes with age. We detected both spontaneous and VSL#3-induced changes in the composition of the rat gut microbiota. Four main bacterial phyla were identified and an age-dependent fluctuation in their relative abundance was documented throughout the study period. At day 0, young and aged rats differed for *Bacteroidetes* composition; this phylum was significantly more abundant in aged rats, while the other bacterial phyla were similar. Interestingly, at day 42, the relative percentage of *Bacteroidetes* increased also in young rats while *Firmicutes* decreased, indicating that bacterial population spontaneously changes during life. These observations support the notion that age represents an important regulatory factor for the gut microbiota both in rats [34] and humans [35], [36].

The data indicate that long-term VSL#3 administration reshapes the intestinal microbiota only in aged rats. The most significant change was an increase in the *Bacteroidetes* and *Actinobacteria* amount in the aged rats treated with VSL#3. This change was comparable to that which occurred spontaneously in young rats whether or not they received VSL#3. Because the *Actinobacteria* phylum includes the *Bifidobacteria* species, one of the main components of VSL#3, these changes suggest that VSL#3 treatment effectively modulates the composition of the intestinal microbiota. Previous studies have shown that VSL#3 promotes a variety of local/intestinal effects including regulation of intestinal permeability and host innate immune response [37]–[42]. In addition to these well-characterized local effects on the gut epithelium, gut immune function and enteric nervous system, long-distance effects of the microbiota on metabolism, liver, adipose tissue and brain have been reported [23].

In the present study, we report that age *per se* resulted in a significantly reshaping of the expression of ∼1% of the entire genoma of rat brain cortex. While the interpretation of modulation in gene expression in the context of complex physiological changes such age is difficult, the bioinformatic approach used in this study to characterize the whole brain rat genome allowed the detection of a well defined signal-to-noise effect on the expression of over 300 genes. Thus, in comparison with young rats, the cortical tissue of aged rats was characterized by upregulation in the expression of 212 genes and downregulation in expression of 111 genes. Among the genes that were upregulated were several that are key in inflammation including Interferon regulatory factor 7 (Irf7), a gene that is significantly downregulated in prefrontal cortex of patients with major depressive disorders [43]. Changes were also observed in GFAP which codes for glial fibrillary acidic protein expressed in astrocytes of central nervous system [44], and phospholipase A2, group III (PLA2G3) that is the highest expressed gene in a neuronal model of oxidative stress induced by the free radical-generating system xanthine/xanthine oxidase [45]. Changes in the expression of PLA2G3 were confirmed by RT-PCR (Figure S1) suggesting that reshaping of this gene is an adaptive change that occurs with age. Importantly PLA2G3 drives apoptotic cell death and its overexpression has been associated with Alzheimer's disease [45]. Changes in the expression of other genes associated with increased risk of developing Alzheimer's disease were also observed including sorting nexin 8 (SNX8) [46] and apolipoprotein D (Apod), which is up-regulated in pathological and stress condition including Alzheimer's disease [47]. Upregulation of oligodendrocyte transcription factor 1 (Olig 1) which controls differentiation and myelin production during inflammation [48] and interferon regulatory factor 9 (Irf9) which is upregulated by neuronal injury [49], suggests that age is associated with resetting of genes that are commonly associated with inflammation [1].

Among the genes that were downregulated in cortical tissue of aged, compared with young, rats were several encoding for proteins related with neuronal development and plasticity. These include nidogen 2 (osteonidogen) (Nid2), a membrane protein expressed in cortex and striatum, whose expression has been reported to decrease with age [50]. This age-related decline in the expression of Nid2 was confirmed in this study by RT-PCR (Figure S1). Interestingly Nid2 prevents aggregation of β amyloid and destabilizes preformed fibrils of β amyloid [50]. Other genes whose expression was negatively modulated by age were the neuromedin B receptor (NMBR) which is highly expressed in area of the brain involved in memory and emotional processing [51] and arachidonate 15-lipoxygenase (Alox15) which is reported to be involved in development of Alzheimer's disease [52].

One important finding of the present study was the observation that VSL#3 treatment resets the expression of a number of genes in the cortex. Thus treatment of aged rats with VSL#3 resulted in the resetting of at least 66 genes of which 25 were upregulated and 41 were downregulated. Significantly, VSL#3 treatment attenuated the age-related changes in 3 genes that impact on inflammation, PLA2G3, Nid2 and Alox15.

Our data agree with previous studies which demonstrated that intestinal microbiota modulates brain gene expression and alters the profiles of canonical signaling pathways, neurotransmitter turnover and synaptic-related proteins which, in turn, influence brain development and function [53]. While changes in the expression of genes in the brain might be functionally relevant, the mechanism involved in their regulation by VSL#3 remains to be determined. One possible mechanism mediating the gut-brain communication may be via established neuronal circuits. Recent data have shown that an impact of probiotics on the brain requires the integrity of the vagus nerve and gut microbiota can elicit signals via the vagal nerve to the brain and *vice versa* [54]. Moreover modulation of transmitters (e.g., serotonin, melatonin, gamma-aminobutyric acid, histamines, and acetylcholine) within the gut is yet another possible mechanism of action that could mediate the effects of the gut microbiota. Alternatively, metagenomics or metatranscriptome studies have demonstrated that ingestion of probiotics impacts on bacterial metabolic activities in the gut redirecting the host metabolism; according to this proposal, changes in microbiota-produced signaling molecules (including amino acid metabolites, short chain fatty acids and neuroactive substances) might be involved [55]. In our study, VSL#3 induced not only a change on gene expression, but also altered the expression of several proteins involved in aging and inflammation, indicating that modulation of these molecules may play a key role in the expression of brain genes.

To evaluate whether the VSL#3-induced changes in gene expression impacted on neuronal function, we assessed LTP in the hippocampus, which has been shown to be adversely affected by age [32], [56]–[61]. LTP is widely used as an indicator of healthy brain function and, accordingly, it is impaired in a number of neurodegenerative disease models which are associated with inflammatory changes. Thus deficits in LTP, accompanied by cognitive dysfunction, have been reported in models of Alzheimer's disease. The present data show that LTP was robustly decreased in aged rats providing further evidence of the negative impact of age on synaptic plasticity. An age-related decrease in cognitive function associated with loss of synaptic plasticity, specifically a deficit in LTP, has been consistently reported [56] and has been attributed variously to dysregulation in calcium handling by cells, altered receptor expression and receptor-mediated signaling, loss of synapses and decreased neurotrophic support [57]. Oxidative and neuroinflammatory changes as a consequence of microglial activation have also been shown to negatively impact on LTP [1]. In the context of neuroinflammatory changes, agents which decrease microglial activation in the brain of aged rats, for example minocycline, atorvastatin, rosiglitazone and polyunsaturated fatty acids, like eicosapentaenoic acid, attenuate the age-related deficit in LTP [32], [58]–[60]. Here we show that VSL#3 attenuates the age-related decrease in LTP. The mechanisms involved in regulation of LTP by VSL#3 are likely to be many; the most parsimonious explanation is that the anti-inflammatory effects of the treatment observed in the gut [61], [62] extend to the brain [63]–[64]. In this context, we provide evidence that VSL#3 modulates hippocampal expression of two markers of microglial activation (and therefore inflammation), CD68 and CD11b, confirming the previously-described inverse correlation between LTP and inflammatory changes. An important observation made in this study is that VSL#3 regulates the expression of specific mediators of synaptic plasticity including BDNF, synapsin and syntaxin in the hippocampus of aged rats. BDNF is essential for maintaining LTP and its role has been long recognized; it's specific function still remains to be clarified [65] though it induces neurogenesis [66] and synaptogenesis [67] and the increased hippocampal expression of BDNF by exercise, has been shown to enhance the ability of aged rats to sustain LTP [68], [69]. BDNF was increased in hippocampus of VSL#3-treated rats and that this was associated with an increase in synapsin, suggesting that VSL#3 exerted a synaptotrophic effect, though the mechanism involved remains to be elucidated.

In conclusion, we have shown that age regulates the expression of several genes in cortical tissue and adversely affected synaptic function. Altering the intestinal microbiota of aged rats by treatment with VSL#3 modulated the expression of a cohort of genes in the cortex, some of which impact on inflammatory processes. We suggest that this effect, together with its neurotrophic/synaptotrophic effect, contributes to the ability of VSL#3 in attenuating the age-related impairment of LTP.

## Supporting Information

Figure S1 Confirmation of microarray data by qRT-PCR analysis (A) Cortical expression of the Nid2 detected by PCR did not change in the four groups of rats. (B-C) PCR confirmed the gene array data for Alox15 and PLA2G3 respectively. *p<0.05 vs Group YC; #p<0.05 vs Group AC.
(TIF)
