# Supplementary figures and images for: Modulation of Intestinal Microbiota by the Probiotic VSL#3 Resets Brain Gene Expression and Ameliorates the Age-Related Deficit in LTP
Source: PLoS One. 2014 Sep 9;9(9):e106503. doi: 10.1371/journal.pone.0106503 (PMC4159266; doi:10.1371/journal.pone.0106503)

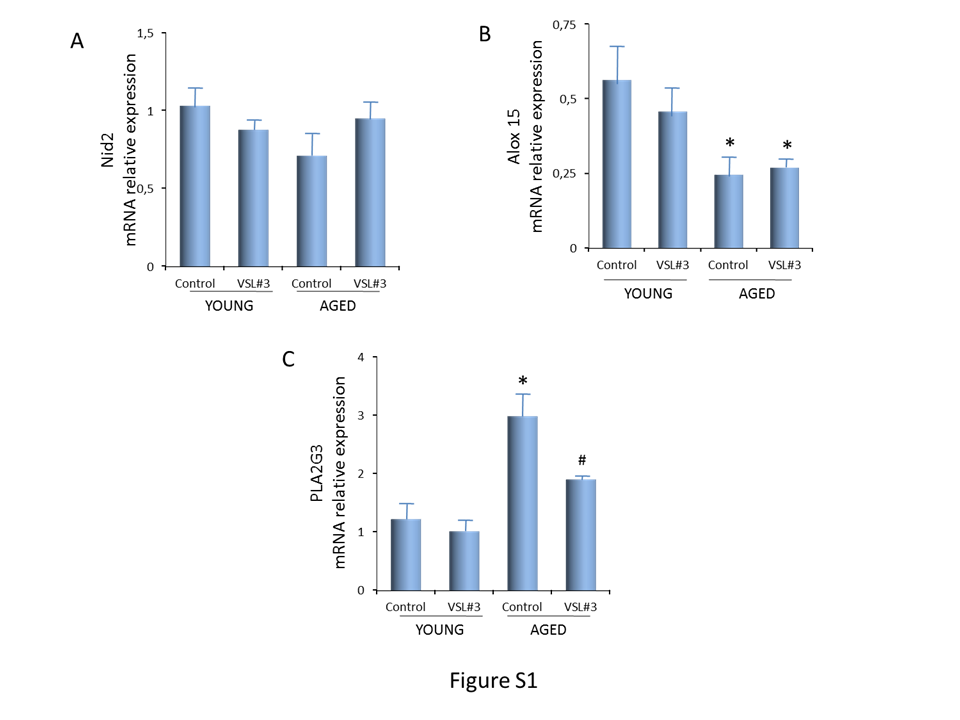

Supplement: Figure S1 — Confirmation of microarray data by qRT-PCR analysis (A) Cortical expression of the Nid2 detected by PCR did not change in the four groups of rats. (B-C) PCR confirmed the gene array data for Alox15 and PLA2G3 respectively. *p<0.05 vs Group YC; #p<0.05 vs Group AC. (TIF) [file pone.0106503.s001.tif]
